# Dual Application of *p*-Nitrophenol Alkanoate-Based Assay for Soil Selection and Screening of Microbial Strains for Bioplastic Degradation

**DOI:** 10.4014/jmb.2403.03013

**Published:** 2024-05-30

**Authors:** Nara Shin, Jinok Oh, Suwon Kim, Yeda Lee, Yuni Shin, Suhye Choi, Shashi Kant Bhatia, Yung-Hun Yang

**Affiliations:** 1 Department of Biological Engineering, College of Engineering, Konkuk University, Seoul 05029, Republic of Korea; 2 Institute for Ubiquitous Information Technology and Application, Konkuk University, Seoul 05029, Republic of Korea

**Keywords:** Soil, esterase assay, screening method, phospholipid fatty acid, polybutylene succinate

## Abstract

With an increase in the commercialization of bioplastics, the importance of screening for plastic-degrading strains and microbes has emerged. Conventional methods for screening such strains are time-consuming and labor-intensive. Therefore, we suggest a method for quickly and effectively screening plastic-degrading microbial strains through dual esterase assays for soil and isolated strains, using *p*-nitrophenyl alkanoates as substrates. To select microbe-abundant soil, the total amount of phospholipid fatty acids (PLFAs) included in each soil sample was analyzed, and esterase assays were performed for each soil sample to compare the esterase activity of each soil. In addition, by analyzing the correlation coefficients and sensitivity between the amount of PLFAs and the degree of esterase activity according to the substrate, it was confirmed that substrate *p*NP-C2 is the most useful index for soil containing several microbes having esterase activity. In addition, esterase assays of the isolated strains allowed us to select the most active strain as the degrading strain, and 16S rRNA results confirmed that it was *Bacillus* sp. N04 showed the highest degradation activity for polybutylene succinate (PBS) as measured in liquid culture for 7 days, with a degradation yield of 99%. Furthermore, *Bacillus* sp. N04 showed degradation activity against various bioplastics. We propose the dual application of *p*-nitrophenyl alkanoates as an efficient method to first select the appropriate soil and then to screen for plastic-degrading strains in it, and conclude that *p*NP-C2 in particular, is a useful indicator.

## Introduction

Synthetic plastics are used in a wide range of human activities and it is difficult, if not impossible, to envisage the modern world without them. One of their distinguishing characteristics is their strong resistance to environmental influences, particularly to biodegradation [1]. However, this property of synthetic plastics is a major cause of environmental pollution [2]. Assuming that the trend of increasing plastic production is maintained, it is predicted that up to 26 billion tons of plastic waste will be generated by 2050. More than half of this waste will be dumped into landfills, eventually flowing into ecological areas such as seas and lakes, leading to serious environmental pollution [3-5]. Bioplastics such as polybutylene succinate (PBS), poly(butyleneadipate-*co*-terephthalate) (PBAT), polylactic acid (PLA), and polyhydroxyalkanoate (PHA) have recently attracted attention as substitutes for petroleum-based synthetic plastics [6]. These bioplastics generate lower greenhouse gas emissions than traditional petroleum-based polymers [7]. This could contribute to a reduction in the overall carbon footprint associated with plastic production [8, 9].

However, along with producing bioplastics, it is also important to degrade them in an appropriate way and the importance of achieving this using microorganisms has recently emerged [10]. Bioplastics degrade into water and carbon dioxide, faster than petroleum-based polymers, via biological processes [11]. Plastics can be degraded in the environment via biodegradation, photolysis, hydrolysis, pyrolysis, or enzymatic methods [12]. The direct use of specific microbes for biodegradation can accelerate bioplastic degradation without additional enzyme synthesis and purification steps [13]. Because microbes can degrade complex polymer structures in plastics into simpler compounds, thereby reducing the persistence of plastic waste in the environment, degrading polymers using microbes seems to be more favorable [14]. In addition, identifying a single strain that can degrade plastic would be an efficient methodology to degrade the target plastic [15].

**Fig. 1:**
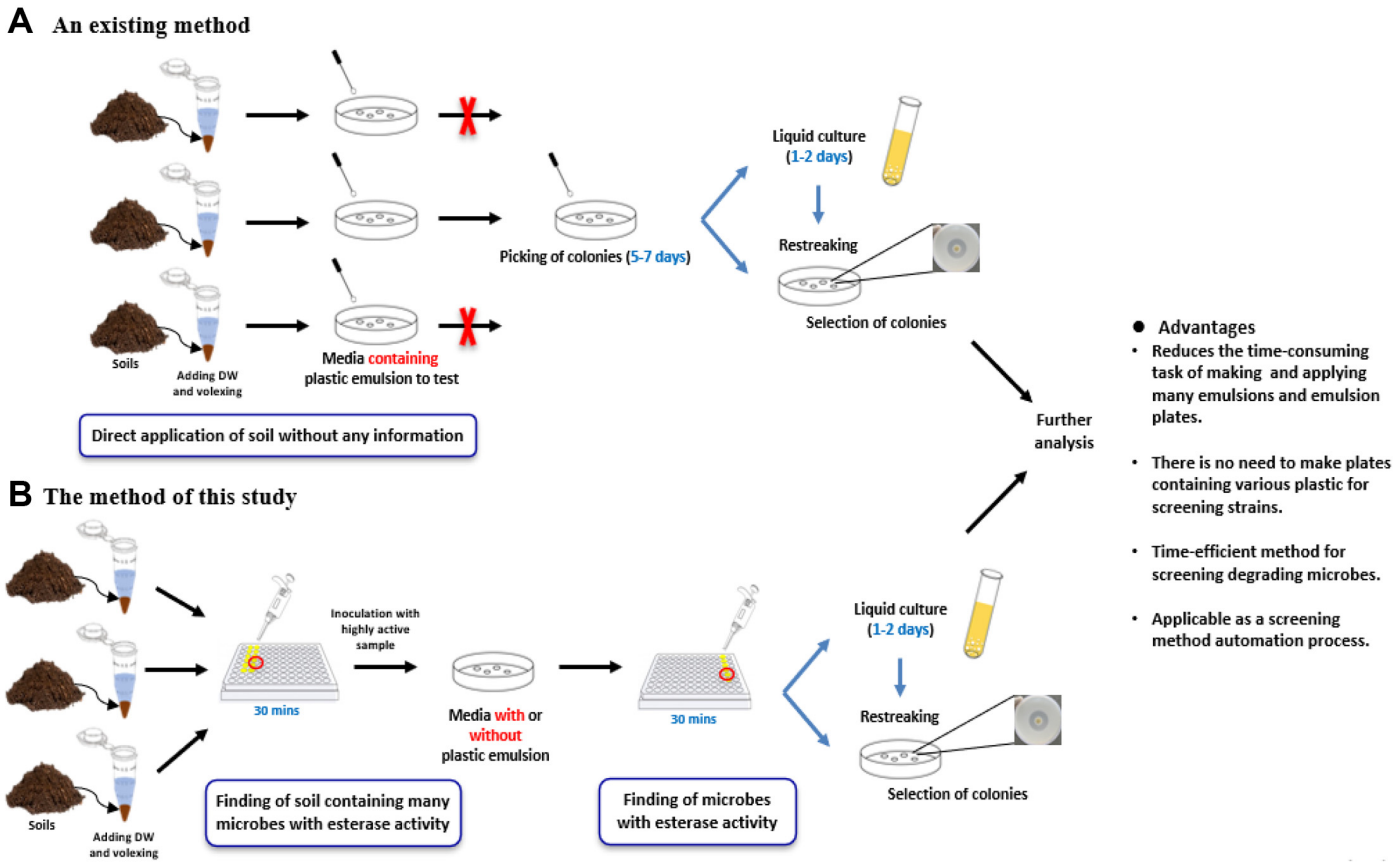
Schemes for soil esterase activity dependent screening process. (**A**) Existing methods for screening plasticdegrading strains. (**B**) New method of screening plastic-degrading strains proposed in this study.

However, the existing method of screening for degradation strains from many microbial sources like soil is lengthy and complicated, involving a series of processes (Fig. 1A). Previously, a solid plate containing a plastic emulsion was prepared to identify the degrading strains, and all soil samples without any information were spread on the plastic plate [16-18]. It takes 5-7 days to spread and pick the colonies. Because there was no information on the soil, all soil samples were spread. After culturing the isolated strains in liquid media for 1-2 days, re-streaking was performed to select colonies that formed clear zones and screen for degradation strains. These conventional degradation strain screening methods are time-consuming and labor-intensive because they require plastic emulsions, and all soil samples must be spread on a plate. Therefore, it is necessary to obtain information about the soil in advance and efficiently select degrading strains that do not require considerable time.

In previous studies, *p*-nitrophenyl ester substrates have been used as indicators of plastic degradation in the soil and to evaluate soil esterase activity (Table 1). In this study, we introduced further applications for screening plastic-degrading strains with dual application of *p*-nitrophenyl alkanoates (Fig. 1B). The method presented here relies on esterase activity evaluation to first identify the soil containing multiple microbes with esterase activity. It makes us select the soil showing significantly higher esterase activity and decrease the number of soil samples to be studied further. Subsequently, the soil sample was spread on a solid medium with or without a plastic emulsion, and the strains were isolated to identify and screen for plastic-degrading strains. The newly suggested screening method has the advantage of reduced duration compared to conventional methods and does not necessarily require the preparation of plates containing various plastic emulsions. Furthermore, this suggests the possibility of using the screening method as an automated process by combining soil selection and strain screening processes using esterase assays. Based on the confirmation of esterase activity in the soil and strains in this study, the newly discovered screening method is expected to greatly contribute to the discovery of microbes that can degrade plastic and solve environmental problems caused by its longevity.

## Materials and Methods

### Chemicals

All chemicals used in this study were of analytical grade. Chloroform and dichloromethane (DCM) were procured from Junsei Chemical Co. (Japan). Polybutylene succinate (PBS) pellets were sourced from ANKOR Bioplastics Co., Ltd. (Republic of Korea). Polycaprolactone (PCL) and polylactic acid (PLA) were purchased from Sigma-Aldrich (USA). Polyhydroxybutyrate (PHB) pellets were obtained from Goodfellow Cambridge Ltd. (UK), and Polybutylene adipate terephthalate (PBAT) was obtained from SK leaveo (Republic of Korea). We obtained *p*-nitrophenyl hexanoate (C6) from Tokyo Chemical Industry, Tokyo, Japan. *p*-nitrophenyl acetate (C2), *p*-nitrophenyl butyrate (C4), *p*-nitrophenyl octanoate (C8), *p*-nitrophenyl decanoate (C10), and *p*-nitrophenyl dodecanoate (C12) were purchased from Sigma.

### Phospholipid Fatty Acid (PLFA) Analysis

To confirm the microbial community structure, phospholipid fatty acid (PLFA) analysis was performed to determine the PLFA patterns in soil microorganisms. All soil samples (Soils A-F) were obtained from ABNEXO Co., Ltd. (Republic of Korea). Following cell membrane dissolution, the lipids were extracted as previously described [19, 20]. The samples were treated with 1 ml of chloroform, 2 ml of methanol, and 1 ml of distilled water (DW). The mixture was then orbitally agitated for 16 h at 25°C. After adding 1 ml of DW and 1 ml of chloroform the mixture was vortexed and then centrifuged at 1,500 ×*g* for 5 min. Then, 2 ml of the liquid phase was transferred to glass vials. The sample was evaporated with N_2_ gas and retreated with 1 ml of chloroform for the following stages. The complete lipid extract was subjected to column chromatography using silicic acid, which binds to lipids. Then, 1 ml of methanol, 1 ml of toluene, and 2 ml of 0.2 M MeOH/KOH were added, the mixture was vortexed, and reacted at 37°C for 20 min. Each lipid in the sample was eluted using a different solvent: chloroform for neutral lipids, acetone for glycolipids, and methanol for phospholipids. Only the phospholipids dissolved in methanol were collected in a glass vial and evaporated using N2 gas. A suspension with 1 ml chloroform was used to prepare final analytical samples.

### Esterase Assay

**Soil esterase activity test.** An esterase test was conducted to identify the soil sample containing multiple microbes with esterase activity. Samples were prepared by adding 1 ml of DW to 0.5 g of soil in a sterile 1.5 ml e-tube, followed by vortexing and incubation at room temperature for 12 h. The supernatant was obtained after centrifugation at 4°C and 13,000 ×*g* for 10 min. The esterase activity of the soil samples was confirmed using six *p*-nitrophenyl esters as substrates: *p*-nitrophenyl acetate, *p*-nitrophenyl butyrate, *p*-nitrophenyl hexanoate, *p*-nitrophenyl octanoate, *p*-nitrophenyl decanoate, and *p*-nitrophenyl decanoate. The enzyme reactions comprised of 180 μl of 50 mM phosphate buffer (pH 7.4), 10 μl of supernatant, 5 μl of ethanol, and 5 μl of substrate dissolved in acetonitrile. Mixtures were reacted in an incubator at 37°C three times every 10 min. Absorbance was measured at 405 nm in a 96-well plate to confirm esterase activity[21]. The absorbance value of each sample containing DW without substrates was used as a control, and the value subtracted from the absorbance value of each sample was taken as the final absorbance value.

**Esterase activity test for cells.** The supernatant containing the esterases secreted outside the cell was used to assess esterase activity. For this, the screened plastic-degrading strains from soil with high esterase activity were precultured and cultured for 16 h at 42°C in tryptic soy broth (TSB) liquid medium in a 15 ml round tube. The bacterial cultures were centrifuged for 10 min at 4°C and 13,000 ×*g* to obtain the supernatants. Six *p*-nitrophenyl esters (C2-C12) used in the soil esterase assay were used as substrates. Prior to the assay, the supernatants were collected from the relevant cell cultures and the cell number was confirmed by measuring the absorbance at 600 nm. The enzymatic reactions contained 180 μl of 50 mM phosphate buffer (pH 7.4), 10 μl of supernatant, 5 μl of ethanol, and 5 μl of substrate dissolved in acetonitrile, and were conducted in an incubator at 37°C three times every 10 min. To confirm esterase activity, absorbance was measured at 405 nm in a 96-well plate.

### Preparation of Plastic Films and Solid Plates Containing Plastics Using Solvent Casting

The preparing PBS films process involved utilizing the solvent-casting. For this, 0.5 g of PBS, PCL, PBAT, PLA, and PHB pellets were dissolved in 250 ml of chloroform and heated in a water bath at 60°C. After the pellets were fully dissolved, the resultant solution was transferred to a Petri dish and placed in a fume hood at room temperature.

To prepare PBS containing plates, 0.5 g of PBS pellets was dissolved in 20 ml of DCM in a water bath at 60°C until the pellet was completely dissolved. This solution was diluted in 50 ml of water, and 1 ml of 2% Sarkosyl NL was introduced at the interface. The resulting mixture was sonicated using a Vibra-Cell VCX500 (Sonics & Materials, Inc., USA) with 15 s of pulsing at 30% amplitude for 10 min. Subsequently, a 1 g/l plastic emulsion uniformly dissolved in the aqueous phase of the solvent was added to marine broth (MB; Difco Laboratories, USA) and 2%agarose. All mixtures were sterilized by autoclaving for 15 min at 121°C

### 16S RNA Ssequencing of the Plastic-Degrading Microbe

The polymerase chain reaction (PCR) comprised 1 μl of template DNA, 10 μl of Hot Start Green mix (consisting of Taq Polymerase, dNTPs, MgCl2, and Buffer), 1 μl of each universal primer 27F (AGA GTT TGA TCM TGG CTC AG) and 1492 R (CGG TTA CCT TGT TAC GAC TT), along with 7 μl of water. The PCR amplification was performed in a LifeTouch thermal cycler (Bioer Technology, China) with the following conditions: initial preheating at 95°C for 3 min, followed by 35 cycles of 95°C for 30 s, 48°C for 39 s, and 72°C for 72 s [22]. Stocks of PBS-degrading microbes were identified at the species level using 16S rRNA gene sequencing (Bionics, Republic of Korea). Sequencing was conducted using the universal primer 27F and partial sequences of each microorganism were aligned against sequences from the National Centre for Biotechnology Information (NCBI) GenBank database using BLASTn.

### Quantification of Degraded Film by Liquid Culture

Using the liquid culture method, films made of PBS, PCL, PBAT, PLA, and PHB were cut into 20 mg pieces and sterilized using 70% ethanol and UV radiation. Then, *Bacillus* sp. N04 cells were inoculated into 5 ml of TSB liquid medium in a 15 ml round tube along with these sterile film fragments. The degradation yield was evaluated at 3, 5, 7, and 10 day intervals during cultivation. We used an inoculum of 2% *Bacillus* sp. N04 cells, and agitated the cultures on a rotary shaker at 200 ×*g*. The remaining film fragments were recovered, washed with DW, and subsequently freeze-dried prior to further experiments. After that, the degradation yield and residual amount analysis of plastics film were performed using GC-MS (YOUNG IN ChroMass, Republic of Korea) using a fused silica capillary column (30 m × 0.25 mm i.d. × 0.25 μm). Prior to this analysis, fatty acid methyl ester derivatization (FAME) was performed to prepare the samples for GC–MS [23, 24]. For methanolysis of plastics film, 1 ml of methanol/sulfuric acid (85:15 v/v) and 1 ml of chloroform mixture were added, and the vials were heated for 120 min at 100°C. The samples were then cooled to room temperature, and 1 ml of HPLC grade water was added and vortexed to mix well. After that, the organic phase layer of the sample was placed in anhydrous sodium sulfate, and the residue was removed. They were then subjected to a linear temperature gradient for analysis. Specifically, this involved an initial 1 min at 50°C, a linear increase of 15°C/min up to 120°C, where it remained for another 2 min; finally, the temperature was increased further at 10°C/min until 300°C, where it remained for 10 min. The injector port temperature was 250°C. Mass spectra were obtained using electron impact ionization at 70 eV, and the scan spectra were obtained within the range of 45–450 m/z. A calibration curve was obtained to estimate the quantity of residual films.

### Monitoring of the Clear Zone by Solid Culture

To analyze and optimize the characteristics of a strain with the ability to degrade PBS, clear-zone tests were performed. *Bacillus* sp. N04 cells were cultured in an optimal liquid medium for 24 h at 42°C to assess the production of a clear zone. Subsequently, 10 μl of the cultured cells were inoculated onto paper discs(Toyo Roshi Kaisha, Japan) positioned on the plate [26, 27], and incubated at 42°C for 14 days. The radius of the clear zones were determined by measuring the distance between the paper disc and the edge of the clear zone. All experiments were conducted in duplicates.

### Analyzing the Physical Characteristics of Films Following Degradation

**Scanning electron microscopy (SEM).** Scanning electron microscopy (SEM) was used to observe surface alterations in each film after degradation. Following *Bacillus* sp. N04-mediated degradation, the residual films were collected, centrifuged, and washed with DW to eliminate medium components before overnight lyophilization. Subsequently, the films were gold-dusted at 5 mA for 300 s and subjected to backscatter electron imaging using a TM4000Plus SEM (Hitachi, Japan) at 5 kV[28].

**Gel permeation chromatography (GPC).** Changes in the molecular weight of the degraded PBS film were examined by gel permeation chromatography (GPC) (YOUNG IN ChroMass). To prepare the samples, the remaining PBS films were dissolved in chloroform and heated at 60°C for 1 h. The resultant solution was filtered using a syringe filter (0.2 μm pore size; Chromdisc, Republic of Korea). The analysis utilized an HPLC apparatus comprising a loop injector (Rheodyne 7725i), an isocratic pump with dual heads (YL9112), a column oven (YL9131), columns (Shodex, K-805, 8.0 mm I.D. × 300 mm; Shodex, K-804, 8.0 mm I.D. × 300 mm), and a refractive index detector (YL9170)., With chloroform serving as the mobile phase and a flow rate of 1.0 ml/min, 60 μl of the sample was analyzed at 35°C. Data were processed using YL-Clarity software designed for a single YL HPLC instrument (YOUNG IN ChroMass). The molecular weight was determined within a range of 5,000 to 2,000,000 g/mol in relation to polystyrene standards [29, 30].

### Statistical Analysis

Data were analyzed using one-way ANOVA in Minitab 18, with the significance threshold set to *P* < 0.05. All experiments were duplicated and expressed as mean ± standard deviation. Principal components analysis (PCA) was used to select a subset of PLFAs important in explaining the variation in the data, and a forward stepwise discriminant analysis was used to determine whether these variables could be used to differentiate the soils. ANOVA in Minitab 18 was used for PCA, and the factor loading scores of individual *p*-nitrophenyl ester substrates were used to evaluate their relative significance in determining the principal component axes.

$$r_{xy}=\frac{\mathrm{cov}(x,y)}{SD_{x}\times SD_{y}}$$

## Results and Discussion

### Difficulties of Selecting Soil based on PBS Films Degradation in Short Time

**Fig. 2:**
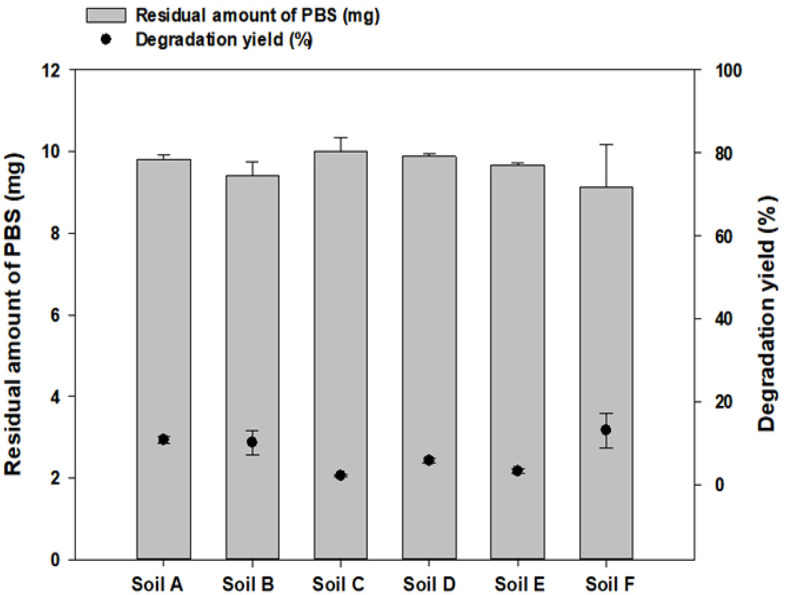
Comparison of PBS degradation yields with each soil sample. Degradation yield after PBS degradation in each soil for 14 days at 42°C was confirmed.

To find out capable microbes degrading bioplastics significantly, the source of microbes is very important. Especially, conducting degradation tests on all available soil samples was deemed laborious and inefficient. As an example in this study to show this, the degradation of PBS films in soil under laboratory conditions was carried out in a 42°C stationary cultivator to select the soil that degrades the PBS well. The tests were conducted for 16 days with 10 mg of PBS film cut into 10 g of each soil sample. Following degradation, the PBS films were recovered from the soil, washed with 70% ethanol, and lyophilized to confirm weight loss. As a result of the degradation test in soils, there was no significant difference in the degradation rate depending on the soils (Fig. 2). Considering that in previous studies, degradation experiments have been conducted for 45-180 days in high-temperature soils [31, 32], short experiments at room temperature did not provide meaningful information about the soil. Furthermore, analyzing the degradation rate after directly collecting the PBS film fragmented from the soil is difficult to accurately quantify due to the degradation film loss. For these reasons, efforts to decrease the number of sources or prioritize experiments are necessary when testing numerous samples. As a result, it is significantly useful to confirm the amount of microorganisms in a short time before starting screening. So far, methods for quantifying the amounts of microorganisms in soil samples can be quantitative real-time polymerase chain reaction (qRT-PCR) and PLFA analysis methods, but qRT-PCR has the problem of amplifying DNA [33]. However, the PLFA analysis method is a useful microbial quantification method that can confirm the total amount of living microorganisms without DNA amplification [34]. It could avoid any amplification issues of samples by monitoring total PLFA from microbes directly with GC-MS.

Since plastic plate preparation is the most difficult bottleneck among the stages of screening bioplastic-degrading strains, we tried to reduce the number of soil samples and save the number of plates for applying plastic plates by prioritizing soil samples with a lot of information such as the number of microorganisms. Therefore, in the next section, after identifying the total amount of microorganisms in the soil within a short time through PLFA analysis, the number of soil sources used in the experiment was reduced or the priority of the experiment was determined by screening the soil with a large total amount of microorganisms.

### PLFA Analysis of Each Soil Sample

PLFA analysis was used to determine the microbial community structure because specific microbial communities have distinct signature fatty acids [35]. Therefore, it was possible to identify changes in the entire soil microbial community based on changes in the PLFA profile [36]. A GC-MS analysis was performed to quantify and compare the amounts of PLFA in each of the six soil samples (Table 2). The PLFA content of Soil A was the highest at 45.59 mg. Soil B contained 39.67 mg and was the second highest after Soil A. Soils C, F, D, and E contained 23.35 mg, 20.82 mg, 12.69 mg, and 12.43 mg of PLFA, respectively. The higher the total PLFA level, the higher the number of living organisms in the soil, confirming that living biomass was the highest in Soil A, followed sequentially by Soils B, C, F, D, and E. Moreover, the PLFA profiles of the entire microbial community revealed a variety of PLFAs in the tested soils, consisting of unsaturated, saturated, methyl-branched, and cyclopropane fatty acids [37]. Saturated/unsaturated (sat/unsat) and Iso/Anteiso ratios can be used as indicators of nutritional imbalance and stress in soil communities [38, 39]. The Iso/Anteiso PLFA ratio was the lowest for Soil A samples and the highest for Soil F samples, indicating that among the six soil samples, the microbial community of Soil A was subjected to lower nutritional stress and the microbial community of Soil F was subjected to greater nutritional stress compared to that of the other soil samples. When comparing the ratio of sat/unsat PLFA as another microbial stress index, it was confirmed that Soil B was the highest at 7.94, and Soil C was the lowest at 1.98.

**Fig. 3:**
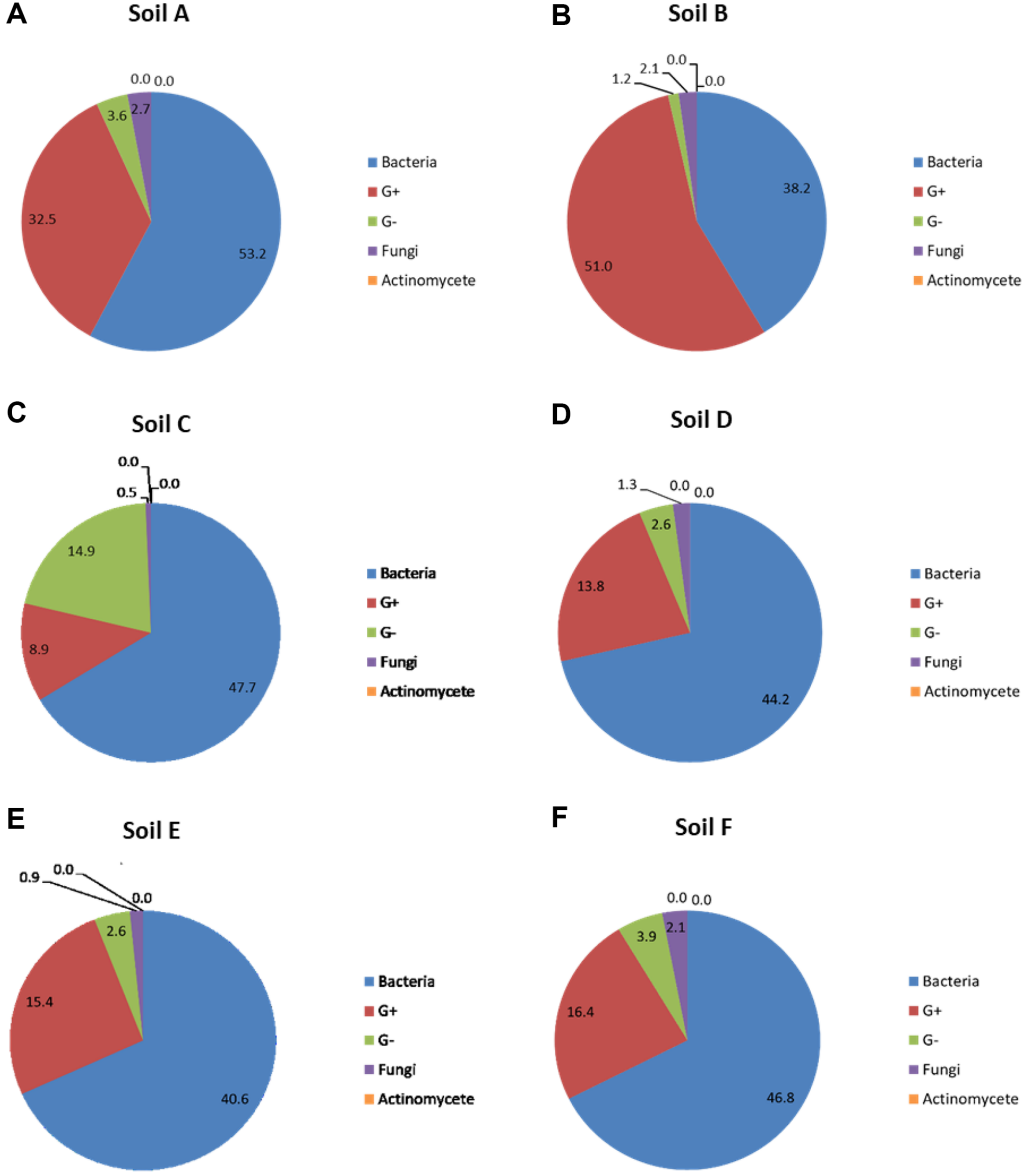
Relative abundances of bacteria, gram-positive bacteria, gram-negative bacteria, fungi, and actinomycete in six soils as determined using phospholipid fatty acid (**PLFA**) analysis. The six soil samples subjected to PLFA analysis were (**A**) Soil A, (**B**) Soil B, (**C**) Soil C, (**D**) Soil D, (**E**) Soil E, and (**F**) Soil F.

Specific PLFAs were used as biomarkers to determine the relative abundances of specific microbial communities in the soil profiles (Fig. 3). Certain lipids can be used to identify the distinct microbial groups. Branched saturated (iso- and anteiso-) lipids, such as i14:0, i15:0, a15:0, i16:0, i17:0, a17:0, and i18:0, have been identified previously as markers of Gram-positive bacteria [40]. The lipids utilized in this study as indicators of Gram-negative bacteria were 16:1w7c, cy-17:0, and cy19:0, as well as saturated fatty acids with an -OH group [41]. Bacterial PLFA were considered as the sum of the marker PLFAs for Gram-positive PLFA, Gram-negative PLFA, and normal saturated fatty acids: C_10:0_, C_12:0_, 14:0, 15:0, a-16:0, 16:0, 17:0, 18:1, C_18:0_, and cy-19:0. Other microbial groups identified with PLFA biomarkers include fungi (18:2ω6c, [42]) and actinomycetes (10Me16:0, 10Me17:0, 10Me18:0, [43]). We identified the proportions of bacteria, Gram-positive bacteria, Gram-negative bacteria, fungi, and actinomycetes in each microbial community through PLFA analysis of each soil sample. When comparing only the relative presence ratio of the microbial community in the soil samples, Soil A had the highest at 53.2% and Soil B had the lowest at 38.2%. For Gram-positive bacteria, Soil B was the highest at 51%, and Soil C was the lowest at 8.9% while for Gram-negative bacteria, Soil C was the highest at 14.9%, and Soil B was the lowest at 2.1%. The relative presence ratio for fungi, was highest in Soil F at 2.1%, and lowest in Soil C at 0.5%. Actinomycetes accounted for 0% in all the soil samples.

Through PLFA analysis, it was confirmed that the total amount of microorganisms in each soil was significantly different and PLFA method clearly gave the hint on which sample should be dealt with first. However, it did not give any information on the high degradation activity of soil and the PLFA analysis was a relatively complex and time-consuming process. Therefore, we initiated to look for another method, such as confirmation of esterase activity in the soil, as PHB, PBS, and PBAT were degraded by esterase [44]. Once esterase activity gives some information like PLFA and additional benefits with a short prescreening time and process, we expect to use it as a screening method for soils to overcome some limitations of soil screening.

### Esterase Assays to Identify Soils with High Esterase Activity

**Fig. 4:**
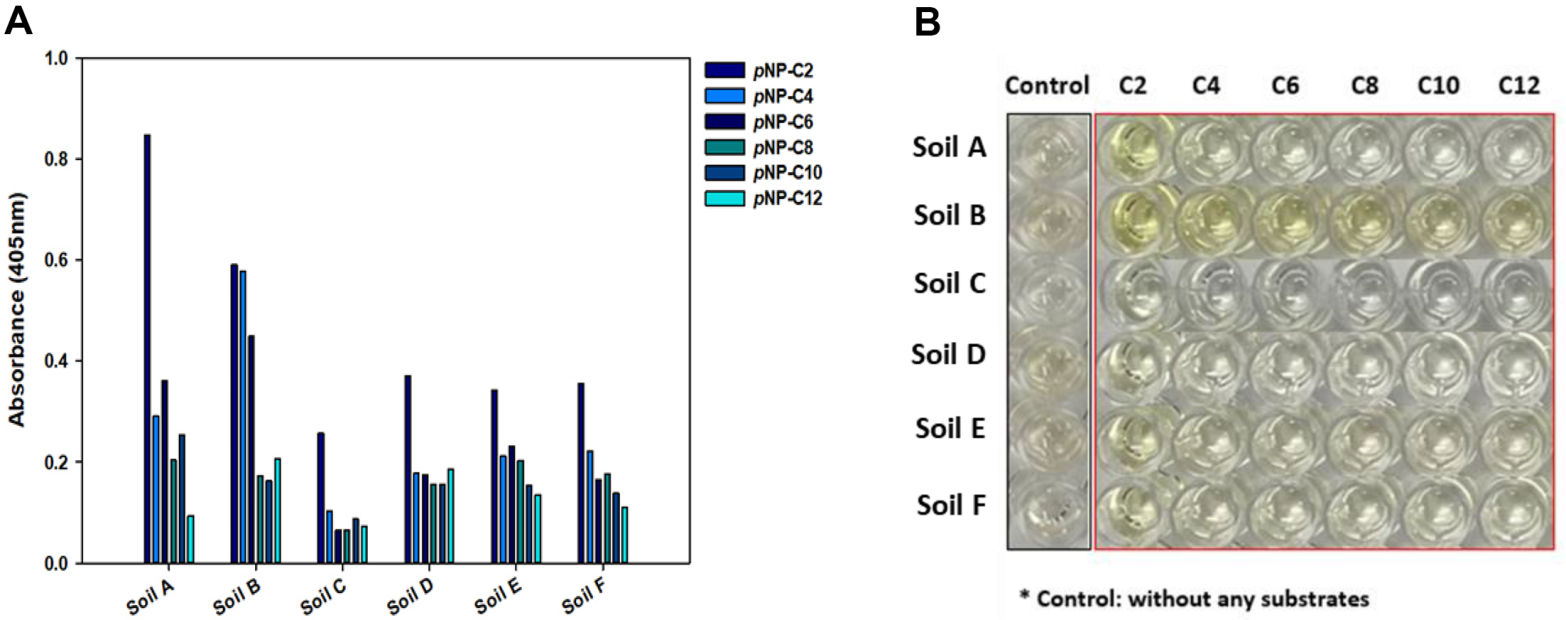
Esterase activity assay for six soil samples. (**A**) Comparing esterase activity of six soils using each supernatant. (**B**) Comparison of color changes to yellow after 30 min of reaction.

To select soils containing numerous microbes with esterase activity, tests were conducted on each soil sample (Fig. 4A). The activity of each substrate (C2-C6) with different chain lengths was evaluated using the supernatant, and excluding the soil. The absorbance values for the substrate *p*NP-C2 measured after 30 min of reaction were 0.848, 0.591, 0.257, 0.371, 0.341, and 0.356 for Soil A, B, C, D, E, and F, respectively. Thus, the activity of Soil A for the substrate *p*NP-C2 was highest, followed by soils B, D, F, E, and C. For substrates *p*NP-C8 and *p*NP-C10, the activity of Soil A was the highest, and for substrates *p*NP-C4, *p*NP-C6, and *p*NP-C12, Soil B had the highest activity. Moreover, the esterase activity of soil samples on the substrate *p*NP-C2 tended to be proportional to the PLFA levels in each soil sample.

To confirm the activity against esterase, the color change to yellow after the reaction was confirmed [45]. After testing esterase activity in 96-well plates, differences in the color change to yellow were observed (Fig. 4B). Each sample had its own color, and Soils B, D, and E were brown to the naked eye. Soil B, for which the visual control was brown and the absorbance value was high; a color change to dark yellow was observed for all substrates. In addition, Soils E and F, which had relatively high absorbance values compared to the control, also showed a yellow color change in all substrates. In the case of Soil C, in which the control color was almost colorless to the naked eye and the absorbance value was the lowest, it was pale yellow with *p*NP-C2, and there was little change in color to yellow in the remaining substrates. In contrast, Soil A showed a color change with substrate *p*NP-C2 to clear and bright yellow, although the color of the control was relatively dark, and the absorbance value of the control was the lowest.

### Correlation Analysis of the Amount of PLFA with Esterase Activity for Each Substrate

**Fig. 5:**
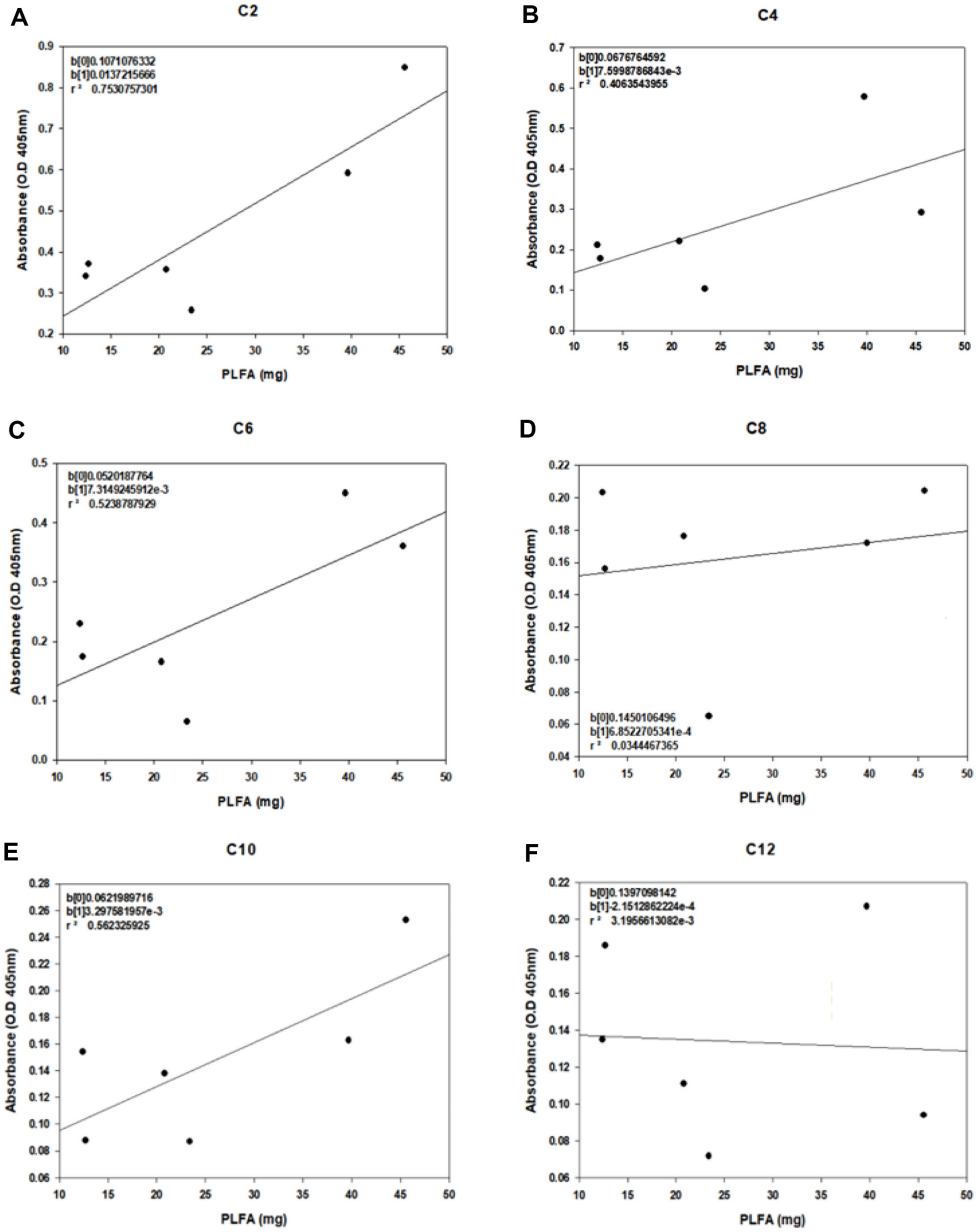
Correlation analysis for soil esterase activity and PLFAs. Regression analysis of the total amount of PLFA in each soil and the absorbance value at 405 nm for a *p*-nitrophenyl ester substrate containing (**A**) *p*NP-C2, (**B**) *p*NP-C4, (**C**) *p*NPC6, (**D**) *p*NP-C8, (**E**) *p*NP-C10, and (**F**) *p*NP-C12.

As a method to identify the soil containing the most microbes with esterase activity, correlation analysis was conducted to determine the most suitable substrate for soil screening when esterase assays were conducted with *p*-nitrophenyl esters as substrates. Therefore, the correlation between the amount of PLFA (mg) in the six soils and the absorbance values for each substrate was analyzed (Fig. 5). The correlation coefficient (r) serves as a metric for gauging the degree of linear connection between two variables [46]. A positive correlation signifies that an increase in one variable corresponds to an increase in another [47]. A correlation of ‘1’ denotes a perfectly positive linear relationship, indicating that the first variable follows the positive linear function of the second variable. Values between zero and these extremes indicate progressively stronger associations in positive and negative relationships, respectively. Typically, a correlation is considered weak if r is ≤ 0.4, moderate if 0.4 < r < 0.8, and strong if r is ≥ 0.8 [48]. Parameters with high correlations were derived using a parameter correlation analysis. When the PLFA (mg) was taken as ‘x’ and the absorbance value as ‘y’, substrate C2 had the highest correlation values for each substrate, followed by *p*NP-C10, *p*NP-C6, *p*NP-C4, *p*NP-C8, and *p*NP-C12. In the correlation analysis, the slope indicates the sensitivity, the larger slope, greater the sensitivity. When the slopes were compared for each substrate, *p*NP-C2 was associated with the largest value, with a slope value of 0.0137, followed by *p*NP-C4 (0.0076), *p*NP-C6 (0.0073), *p*NP-C10 (0.0024), *p*NP-C8 (0.0007), and *p*NP-C12 (-0.0002). Based on the comparison of the correlation coefficients and sensitivity, the substrate with the highest correlation and sensitivity to the amount of PLFA in the soil was *p*NP-C2. These results indicated that substrate *p*NP-C2 is useful indicator for the analysis of esterase activity to screen soil containing many microorganisms with esterase activity and furthermore to screen degradation strains using that soil.

A principal component analysis (PCA) was performed for discriminant analysis to determine whether *p*-nitrophenyl ester substrates could be used to distinguish soils with high esterase activity (Fig. S1). For *p*NP-C2, the PC1 axis represented 97% of all data features, with the two higher samples Soil A and Soil B, having high optical density values based on the PC1 axis, while the four lower samples, Soils C, F, D, and E, clustered. This analysis indicated that *p*NP-C2 among the substrates shares a higher level of similarity with the PLFA results compared to other substrates.

### Screening of PBS Degrading Strains Using Esterase Assay

To show the further step of soil screening, we actually screened PBS degrading bacteria from Soil A. As Soil A was found to contain the maximum number of microbes containing esterases, it was chosen for the isolation of the degradation strains. The finding of PBS degrading bacteria might be an issue of fortune, however, we wanted to show how the esterase activity was applied to select soil and how selected soil could be actually used to screen PBS degrading bacteria. In addition, we want to test the possibilities of an automatic process in the future with fewer labors and a short time to final selection of PBS degrading bacteria. Although the best soil was selected here, the avoidance of low-activity soil seemed useful later.

**Fig. 6:**
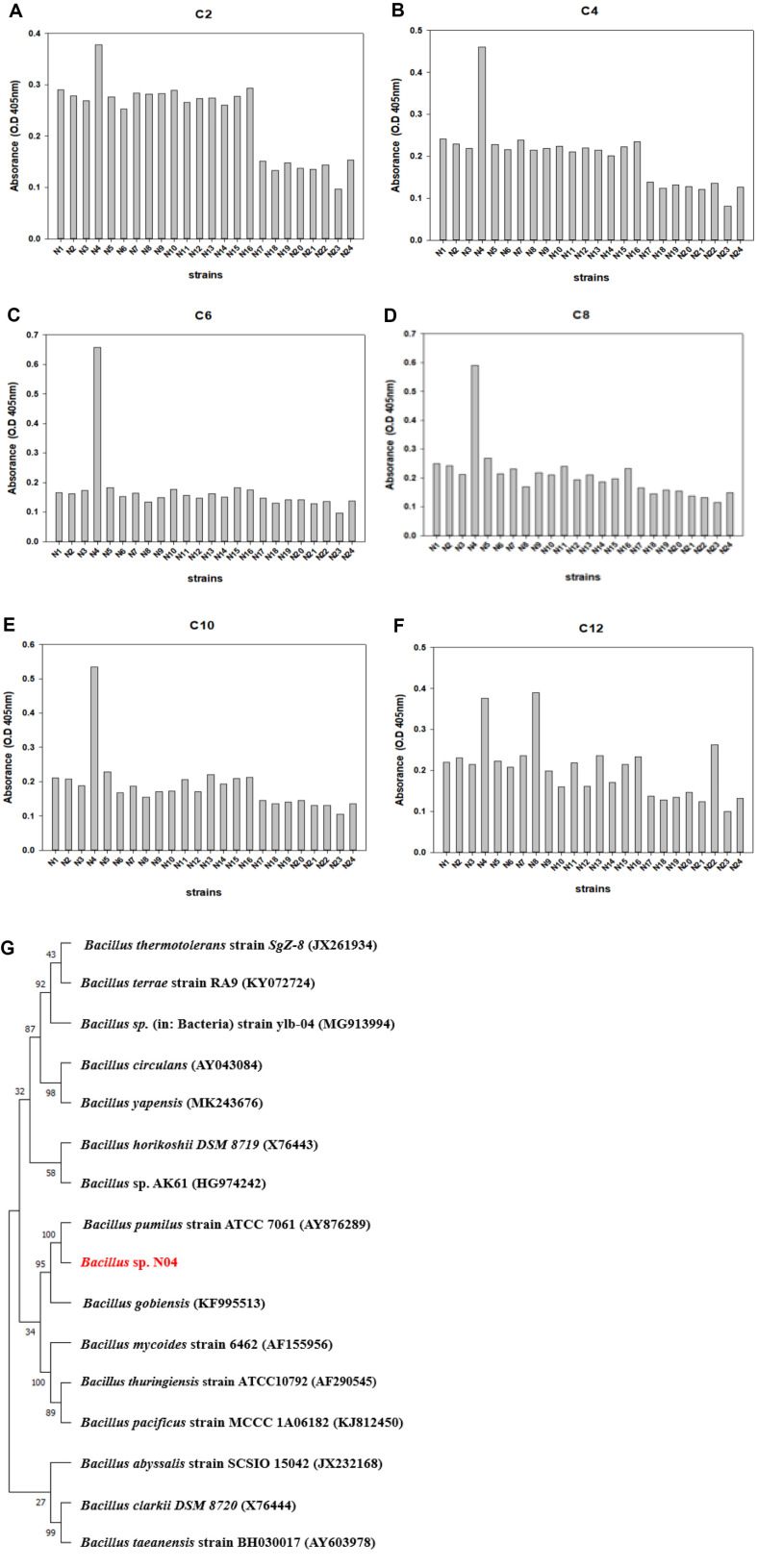
Comparison of esterase activity for strains isolated from Soil A. Esterase activity analysis of strains isolated from Soil A was conducted using *p*-nitrophenyl ester substrate containing (**A**) *p*NP-C2, (**B**) *p*NP-C4, (**C**) *p*NP-C6, (**D**) *p*NP-C8, (**E**) *p*NP-C10, and (**F**) *p*NP-C12. (**G**) Phylogenetic tree of *Bacillus* sp. N04 according to 16S rRNA sequencing.

To find PBS degrading bacteria, soil sample A was spread onto solid TSB plates. After incubation in a 42°C stationary incubator for 2 days, twenty-four different microorganisms (N01-N24) were isolated. To screen for microbes with high esterase activity among these, we checked esterase activities upon the individual addition of substrates of different lengths to each of the strains[49]. The assay was performed on 96-well plates at 10 min intervals, and the absorbance was measured at 405 nm. After the reaction, the wells turned bright yellow owing to extracellular esterase enzyme activity. When the esterase activity of each microbe was compared for the six substrates C2-C12, *Bacillus* sp. N04 showed the highest esterase activity with all the substrates, except *p*NP-C12 (Fig. 6A-6F).

16S rRNA sequencing was used to construct a phylogenetic tree for *Bacillus* sp. N04 to determine the evolutionary relationships within this genus. Using this sequencing method, we found that N04 had the highest similarity to *Bacillus pumilus* (98.63%) (Fig. 6G).

### Time-Dependent Monitoring of PBS Degradation by *Bacillus* sp. N04

**Fig. 7:**
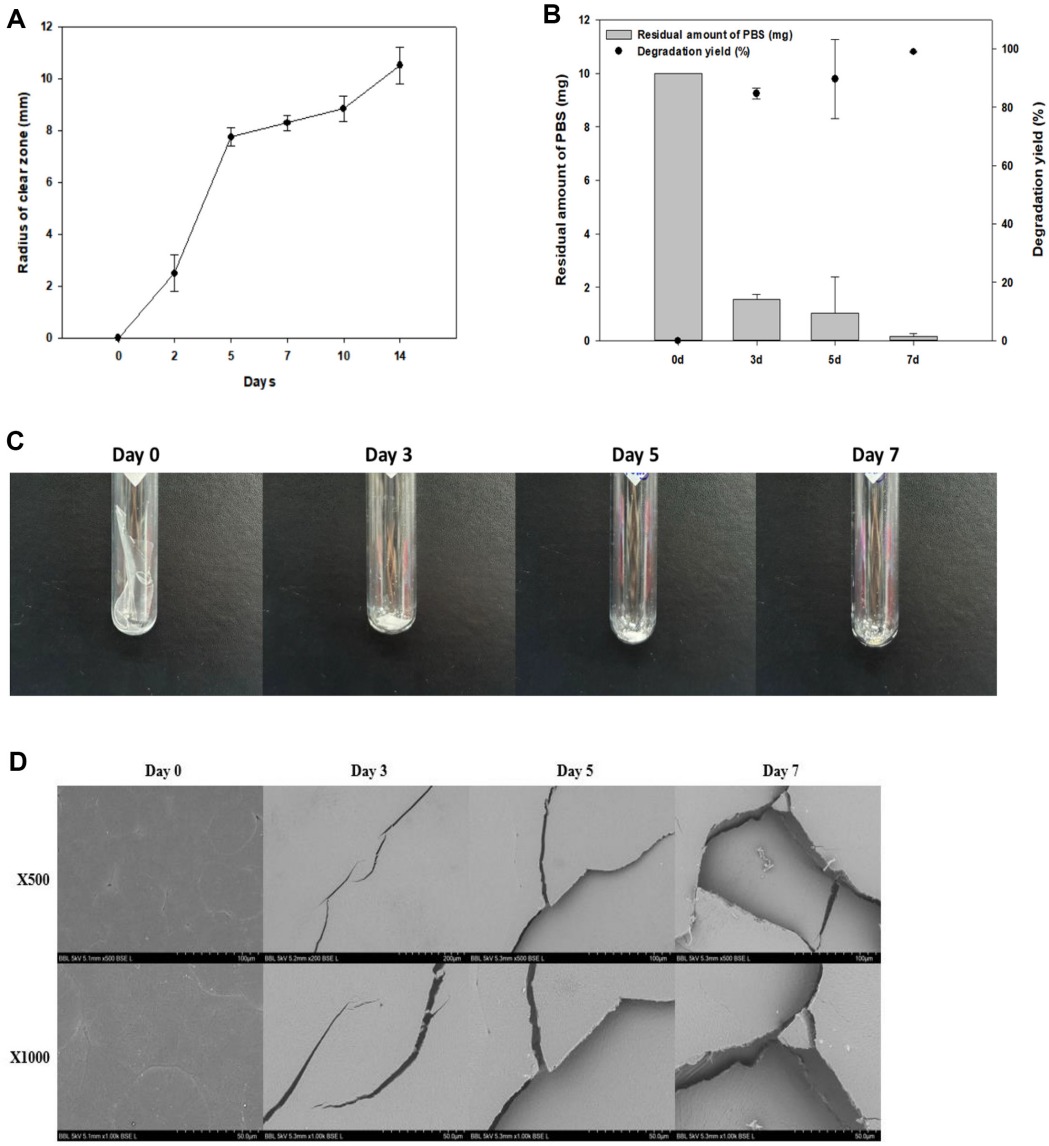
Confirmation of PBS degradation by *Bacillus* sp. N04 over the incubation period. (**A**) Clear zone test for 14 days on solid medium containing PBS plastic emulsion in TSB. (**B**) GC-MS results for degradation yield of PBS according to degradation time (days 0, 3, 5, and 7). (**C**) The visual appearance of PBS films undergoes alterations as a result of degradation. Changes in the degradation of PBS films are observed at different incubation periods (0, 3, 5, and 7 days). Representative images of surface changes observed by (**D**) scanning electron microscopy (SEM).

To confirm the PBS degradation activity of *Bacillus* sp. N04, a clear zone test was performed on TSB solid medium with 1% PBS emulsion at 42°C for 14 days (Fig. 7A). On day 2 of incubation, the radius of the clear zone was approximately 2.23 mm, while it was 10.9 mm on the 14th day. As the incubation time increased, the radius of the clear zone also increased and the zone became transparent.

We examined the patterns of deterioration in the liquid cultures at different time points. The PBS degradation yield of *Bacillus* sp. N04 changed over time (Days 0, 3, 5, and 7). In this experiment, we added 20 mg films to the TSB liquid medium and conducted the experiment at 42°C. The degradation yield of PBS increased with culture time on days 3, 5, and 7. PBS degradation yields were 84.7% on day 3 and 89.6% on day 5. At the end of day 7, the PBS degradation yield reached 99% (Fig. 7B). The degradation of PBS by microorganisms occurs when microorganisms metabolize the chemical bonds of polymers as sources of energy or nutrients, or by secretory enzymes [50]. PBS is a biodegradable aliphatic polyester with the ability of several hydrolytic enzymes, such as lipase, esterase, cutinase, and protease, to catalyze the hydrolysis of various aliphatic polyester [51-53]. These microorganism-secreted enzymes degradation large PBS polymer chains into small fragments, such as oligomers and monomers [54]. The microorganism then metabolizes these small molecules again, eventually turning them into simpler organic compounds such as CO_2_, H_2_O, and biomass [55].

As the PBS film was degraded, its physical properties changed. To evaluate these changes, a PBS degradation test in liquid culture was performed. The PBS films were analyzed using SEM and GPC to assess surface changes and molecular weight alterations respectively, during degradation by *Bacillus* sp. N04. PBS degradation by *Bacillus* sp. N04 lasted 7 days, and changes in morphology were observed following the lyophilization of the PBS films that remained after degradation on days 0, 3, 5, and 7 (Fig. 7C). On day 3 of degradation, it was confirmed that the amount of each PBS film had degraded compared to that before degradation. On day 5, the films were split more than on day 3, and the PBS was considerably degraded. On day 7, continued degradation resulted in the films was almost completely degraded and disappeared. SEM analysis showed the film surface change with degradation. Before degradation, the surface of the PBS film was smooth. After the 3rd day, multiple small cracks began to form on the surface of the film; from day 5, the crack size increased, and other cracks began to form around the initial ones. On day 7, the holes caused by the cracks became larger and more numerous (Fig. 7D).

GPC analysis revealed differences in the weight-average molecular weight (M_w_), number-average molecular weight (M_n_), and polydispersity index (PDI) of the PBS films, following degradation for 7 days (Table 3). As the PBS films degraded, their M_w_ decreased from 3.68 × 10^4^ to 1.92 × 10^4^. These results indicate that *Bacillus* sp. N04 cleaves long-chain PBS molecules into lower molecular weight fragments. The M_n_ value also decreased from 2.87 × 10^4^ to 2.12 × 10^4^. The PDI was measured to determine the M_w_ distribution of the polymer [56]. The PDI value of the control was 2.47; this value increased to 7.63 on day 3 of PBS film degradation and decreased again to 2.50 on day 5. On day 7 of degradation, the PDI decreased slightly to 2.49. Except on day 3, the PDI value did not change significantly. This lack of difference is consistent with the observations in previous studies on PBS [18]. However, the PDI values differed slightly, depending on the polymer used. In the case of *Microbulbifer* sp. SOL03-mediated degradation of PHB, GPC findings from a previous study showed that PHB had a greater reduction in molecular weight than PBS, and the PDI value changed significantly as degradation proceeded [16].

### Degradability of Various Plastics by *Bacillus* sp. N04

**Fig. 8:**
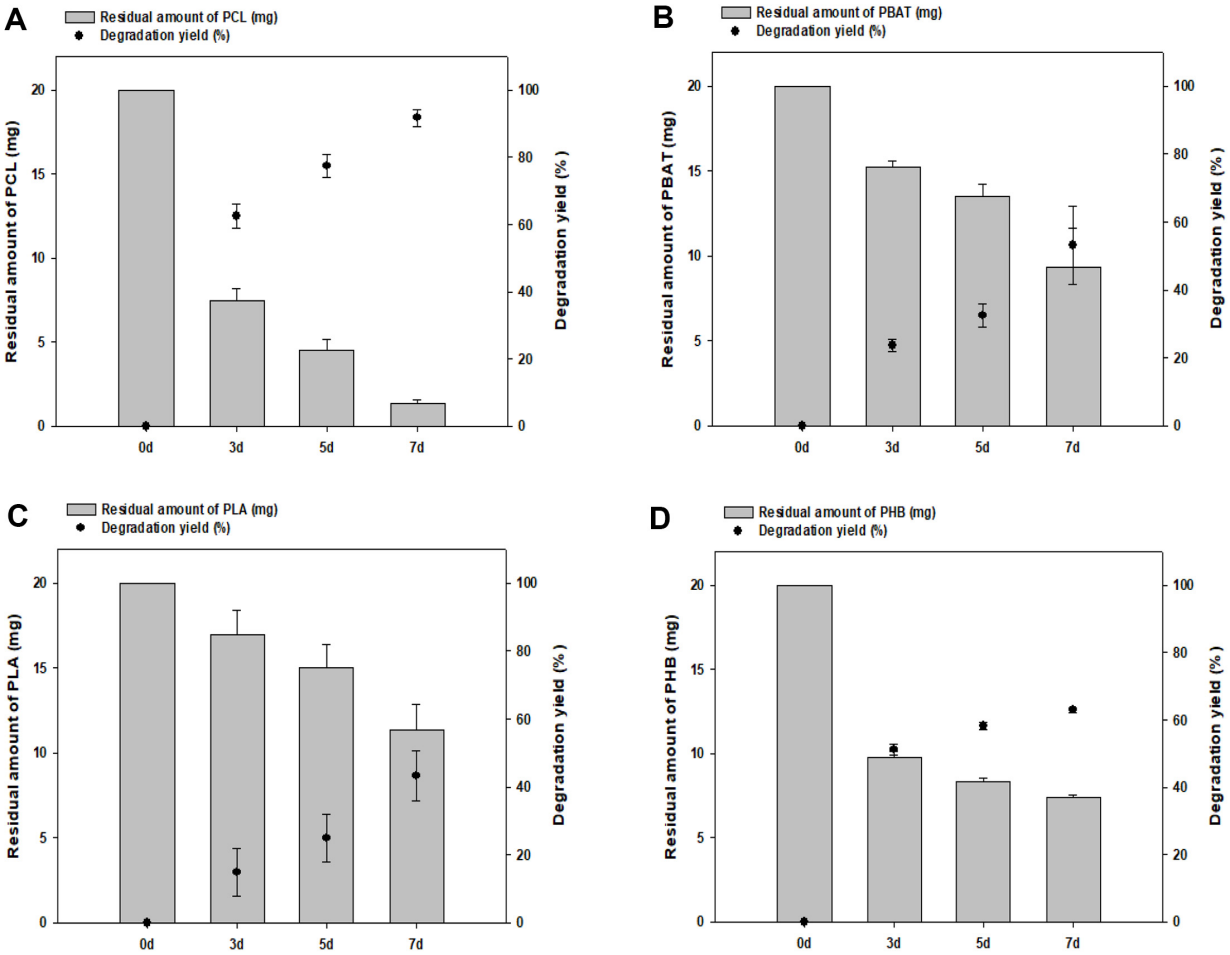
Ability of *Bacillus* sp. N04 to degrade various plastics. The degradation potential of *Bacillus* sp. N04 for several plastics containing (**A**) PCL, (**B**) PBAT, (**C**) PLA, and (**D**) PHB was confirmed for 7 days at 42°C in TSB liquid medium.

To confirm the ability of *Bacillus* sp. N04 to degrade various plastics, the degradation of PCL, PBAT, PLA, and PHB was evaluated. When *Bacillus* sp. N04 cells were incubated with each plastic in TSB liquid medium at 42°C for 7 days, a degradation yield of approximately 95% for PCL and 56% for PBAT were obtained (Fig. 8A and 8B). Furthermore, PLA was degraded by approximately 42% and PHB by approximately 62% (Fig. 8C and 8D). Our findings further indicate that *Bacillus* sp. N04 has the potential to degrade not only PBS but also other aliphatic polymers, including PLA, which is relatively difficult to degrade.

### Conclusion

With the increasing commercialization of bioplastics, an environmentally friendly alternative to traditional petroleum-based synthetic plastics, microbe-mediated plastic degradation is gaining attention as an effective method; accordingly, it is important to efficiently screen for degrading strains. In this study, a fast and efficient screening method was proposed using a dual esterase assay with *p*-nitrophenyl alkanoate as a substrate. This method compensated for the limitations of the conventional method of screening degrading strains, which is time-consuming and labor-intensive due to the relatively complex process. It could significantly decrease the number of soil samples to be tested and give experimental priority. Through a comprehensive analysis, it was confirmed that *p*NP-C2 was the most reliable indicator for evaluating esterase activity in the soil and isolated strains. Upon analyzing the plastic-degrading strains identified through the new screening method and followed by 16S rRNA sequencing fortunately, we confirmed it to be *Bacillus pumilus* (*Bacillus* sp. N04). *Bacillus* sp. N04 showed remarkable plastic degradation ability, especially high activity against PBS, with a decomposition yield of 99% over 7 days. Using SEM and GPC, the changes in the surface and molecular weight due to PBS degradation were confirmed. In addition, this strain exhibited degradation activity across a spectrum of plastics including PCL, PBAT, PLA, and PHB. The proposed dual application of *p*-nitrophenyl could be an efficient strategy for screening plastic-degrading strains, with *p*NP-C2 serving as a useful indicator. The correlation between the total number of microorganisms in the soil and esterase activity does not necessarily align with the degradation of bioplastics; nonetheless, it demonstrates a remarkable correlation. Although these new screening methods may not guarantee the best screening for bioplastic degradation among several soils, it is possible to evaluate the presence of microorganisms with soil esterase activity and to select soils with low activity in the process of discovering other bioplastic-degrading strains. This study contributes to the development of practical and time-efficient methods to identify potent plastic-degrading microbes, especially with esterase activity, thereby providing promising insights into the sustainable management of plastic waste. Furthermore, we expect that this new degrading strain screening method can be utilized as a screening technique for automated processes.

## Supplemental Materials

Supplementary data for this paper are available on-line only at http://jmb.or.kr.



## Figures and Tables

**Table 1:** Previous studies of esterase activity tests on soil for plastic degradation.

Subject to applicable	Substrate as indicator	Degrading Plastic	Reference
Biodegradation detection of poly(butylene succinic acid) aliphatic polyester in soil.	*p*NP-C2	PBS	[57]
Evaluation soil esterase activity using *p*-nitrophenyl valerate	*p*NP-C4	-	[58]
Biodegradation detection of biodegradable polyester mulch film in soil.	*p*NP-C4	PBSA	[59]
Detection of degree of plastic film degradation according to distribution ratio of PBSA-degrading fungi in soil.	*p*NP-C5	PBSA	[60]
Selecting good soil and screening high degrading strain	*p*NP-C2	PBS, PCL, PBAT, PLA, PHB	In this study

**Table 2:** Phospholipid fatty acid (PLFA) analysis of soil samples.

	Soil A	Soil B	Soil C	Soil D	Soil E	Soil F
PLFA (mg)	45.59	39.67	23.35	12.69	12.43	20.82
Sat/unsat	3.43	7.94	1.98	2.04	2.81	2.12
Iso/Anteiso	1.05	1.52	1.38	1.97	2.03	2.17

**Table 3:** Changes in molecular weight of PBS as it is degraded by *Bacillus* sp. N04.

Days	*M_w_* × 10^4^	*M_n_* × 10^4^	*M_w_*/*M_n_* (PDI)
Control	3.68	2.87	2.47
3 days	2.29	2.57	7.63
5 days	2.08	2.39	2.50
7 days	1.92	2.12	2.49

## References

[1] Kotova IB, Taktarova YV, Tsavkelova EA, Egorova MA, Bubnov IA, Malakhova DV (2021). Microbial degradation of plastics and approaches to make it more efficient. Microbiology (Russian Federation).

[2] Alshehrei F (2017). Biodegradation of synthetic and natural plastic by microorganisms. J. Appl. Environ. Microbiol.

[3] Rochman CM, Hoh E, Kurobe T, Teh SJ (2013). Ingested plastic transfers hazardous chemicals to fish and induces hepatic stress. Sci. Rep..

[4] Kibria MG, Masuk NI, Safayet R, Nguyen HQ, Mourshed M (2023). Plastic waste: challenges and opportunities to mitigate pollution and effective management. Int. J. Environ. Res..

[5] Geyer R, Jambeck JR, Law KL (2017). Production, use, and fate of all plastics ever made. Sci. Adv..

[6] Paul S, Sen B, Das S, Abbas SJ, Pradhan SN, Sen K (2023). Incarnation of bioplastics: recuperation of plastic pollution. Int. J. Environ. Anal. Chem..

[7] Selvamurugan Muthusamy M, Pramasivam S (2019). Bioplastics - An eco-friendly alternative to petrochemical plastics. Curr. World Environ..

[8] Bishop G, Styles D, Lens PNL (2021). Environmental performance comparison of bioplastics and petrochemical plastics: a review of life cycle assessment (LCA) methodological decisions. Resour. Conserv. Recycl..

[9] Brizga J, Hubacek K, Feng K (2020). The unintended side effects of bioplastics: carbon, land, and water footprints. One Earth.

[10] Urbanek AK, Rymowicz W, Mirończuk AM (2018). Degradation of plastics and plastic-degrading bacteria in cold marine habitats. Appl. Microbiol. Biotechnol..

[11] Shlush E, Davidovich-Pinhas M (2022). Bioplastics for food packaging. Trends Food Sci. Technol..

[12] Chamas A, Moon H, Zheng J, Qiu Y, Tabassum T, Jang JH (2020). Degradation rates of plastics in the environment. ACS Sustain. Chem. Eng..

[13] Folino A, Karageorgiou A, Calabrò PS, Komilis D (2020). Biodegradation of wasted bioplastics in natural and industrial environments: a review. Sustainability (Switzerland).

[14] Shah AA, Hasan F, Hameed A, Ahmed S (2008). Biological degradation of plastics: a comprehensive review. Biotechnol. Adv..

[15] Taghavi N, Singhal N, Zhuang WQ, Baroutian S (2021). Degradation of plastic waste using stimulated and naturally occurring microbial strains. Chemosphere.

[16] Park SL, Cho JY, Kim SH, Bhatia SK, Gurav R, Park SH (2021). Isolation of *microbulbifer* sp. Sol66 with high polyhydroxyalkanoatedegrading activity from the marine environment. Polymers (Basel).

[17] Cho JY, Park SL, Kim SH, Jung HJ, Cho DH, Kim BC (2022). Novel poly (butylene adipate-*co*-terephthalate)-degrading *Bacillus* sp. JY35 from wastewater sludge and its broad degradation of various bioplastics. Waste Manag..

[18] Kim SH, Cho JY, Cho DH, Jung HJ, Kim BC, Bhatia SK (2022). Acceleration of polybutylene succinate biodegradation by *Terribacillus* sp. JY49 isolated from a marine environment. Polymers (Basel).

[19] Choi TR, Oh SJ, Hwang JH, Kim HJ, Shin N, Yun J (2023). Direct monitoring of membrane fatty acid changes and effects on the isoleucine/valine pathways in an ndgR deletion mutant of *Streptomyces coelicolor*. J. Microbiol. Biotechnol..

[20] Frostegård Å, Tunlid A, Bååth E (2011). Use and misuse of PLFA measurements in soils. Soil Biol. Biochem..

[21] Yun HJ, Lee YJ, Yeo SH, Choi HS, Park HY, Park HD (2012). The isolation and culture characterization of a lipolytic enzyme producing strain from Meju. Korean J. Microbiol. Biotechnol..

[22] Biki SP, Mahmud S, Akhter S, Rahman MdJ, Rix JJ, Al Bachchu MdA (2021). Polyethylene degradation by *Ralstonia* sp. strain SKM2 and *Bacillus* sp. strain SM1 isolated from land fill soil site. Environ. Technol. Innov..

[23] Sathiyanarayanan G, Bhatia SK, Song HS, Jeon JM, Kim J, Lee YK (2017). Production and characterization of medium-chainlength polyhydroxyalkanoate copolymer from Arctic psychrotrophic bacterium *Pseudomonas* sp. PAMC 28620. Int. J. Biol. Macromol..

[24] Bhatia SK, Gurav R, Choi TR, Han YH, Park YL, Park JY (2019). Bioconversion of barley straw lignin into biodiesel using *Rhodococcus* sp. YHY01. Bioresour. Technol..

[25] Choi TR, Park YL, Song HS, Lee SM, Park SL, Lee HS (2021). Fructose-based production of short-chain-length and mediumchain-length polyhydroxyalkanoate copolymer by arctic *Pseudomonas* sp. B14-6. Polymers (Basel).

[26] Bhatia SK, Yoon JJ, Kim HJ, Hong JW, Gi Hong Y, Song HS (2018). Engineering of artificial microbial consortia of *Ralstonia eutropha* and *Bacillus subtilis* for poly(3-hydroxybutyrate-*co*-3-hydroxyvalerate) copolymer production from sugarcane sugar without precursor feeding. Bioresour. Technol..

[27] Shin N, Kim SH, Cho JY, Hwang JH, Kim HJ, Oh SJ (2023). Fast degradation of polycaprolactone/poly (butylene adipate-*co*-terephthalate) blends by novel *Bacillus* strain NR4 with broad dgrading activity. J. Polym. Environ..

[28] Jung HR, Choi TR, Han YH, Park YL, Park JY, Song HS (2020). Production of blue-colored polyhydroxybutyrate (PHB) by onepot production and coextraction of indigo and PHB from recombinant *Escherichia coli*. Dyes Pigm..

[29] Ashby RD, Solaiman DKY (2008). Poly (hydroxyalkanoate) biosynthesis from crude alaskan pollock (theragra chalcogramma) oil. J. Polym. Environ..

[30] Kulkarni SO, Kanekar PP, Jog JP, Patil PA, Nilegaonkar SS, Sarnaik SS (2011). Characterisation of copolymer, poly (hydroxybutyrate-co-hydroxyvalerate) (PHB-*co*-PHV) produced by *Halomonas campisalis* (MCM B-1027), its biodegradability and potential application. Bioresour. Technol..

[31] Pischedda A, Tosin M, Degli-Innocenti F (2019). Biodegradation of plastics in soil: the effect of temperature. Polym. Degrad. Stab..

[32] Huang D, Xu Y, Lei F, Yu X, Ouyang Z, Chen Y (2021). Degradation of polyethylene plastic in soil and effects on microbial community composition. J. Hazard. Mater..

[33] Zhang T, Fang HHP (2006). Applications of real-time polymerase chain reaction for quantification of microorganisms in environmental samples. Appl. Microbiol. Biotechnol..

[34] Zelles L (1999). Fatty acid patterns of phospholipids and lipopolysaccharides in the characterisation of microbial communities in soil: a review. Biol. Fertil. Soils.

[35] Fernandes MF, Saxena J, Dick RP (2013). Comparison of whole-cell fatty acid (MIDI) or phospholipid fatty acid (PLFA) extractants as biomarkers to profile soil microbial communities. Microb. Ecol..

[36] Xue D, Yao HY, Ge DY, Huang CY (2008). Soil microbial community structure in diverse land use systems: a comparative study using biolog, DGGE, and PLFA analyses. Pedosphere.

[37] Zhang Q Chun, Wang G Huo, Yao H Ying (2007). Phospholipid fatty acid patterns of microbial communities in paddy soil under different fertilizer treatments. J. Environ. Sci..

[38] McKinley VL, Peacock AD, White DC (2005). Microbial community PLFA and PHB responses to ecosystem restoration in tallgrass prairie soils. Soil Biol. Biochem..

[39] Bach EM, Baer SG, Meyer CK, Six J (2010). Soil texture affects soil microbial and structural recovery during grassland restoration. Soil Biol. Biochem..

[40] Zhang H, Zhang X, Zhao J, Du X, Ma B (2016). Analysis of the microbial communities of three kinds of Fen-Daqu by PLFAs. J. Inst. Brew..

[41] Xiao L, Liu G bin, Xue S, Zhang C (2013). Soil microbial community composition during natural recovery in the loess plateau, China. J. Integr. Agric..

[42] Cahyani VR, Watanabe A, Asakawa S, Kimura M, Matsuya K (2002). Succession of microbiota estimated by phospholipid fatty acid analysis and changes in organic constituents during the composting process of rice straw. Soil Sci. Plant Nutr..

[43] Zhao Z, Ge T, Gunina A, Li Y, Zhu Z, Peng P (2019). Carbon and nitrogen availability in paddy soil affects rice photosynthate allocation, microbial community composition, and priming: combining continuous 13C labeling with PLFA analysis. Plant Soil.

[44] Santos-Beneit F, Chen LM, Bordel S, Frutos de la Flor R, García-Depraect O, Lebrero R (2023). Screening enzymes that can depolymerize commercial biodegradable polymers: heterologous expression of *Fusarium solani* cutinase in *Escherichia coli*. Microorganisms.

[45] Kim SH, Shin N, Jeon JM, Yoon JJ, Joo JC, Kim HT (2024). Application of liquid-based colorimetric method for high throughput screening of bioplastic-degrading strains using esterase assay. Anal. Biochem..

[46] Asuero AG, Sayago A, González AG (2006). The correlation coefficient: an overview. Crit. Rev. Anal. Chem.

[47] Schober P, Schwarte LA (2018). Correlation coefficients: appropriate use and interpretation. Anesth. Analg..

[48] Shi R, Conrad SA (2009). Correlation and regression analysis. Annals Allergy, Asthma Immunol..

[49] Tarno H, Qi H, Endoh R, Kobayashi M, Goto H, Futai K (2011). Types of frass produced by the ambrosia beetle *Platypus quercivorus* during gallery construction, and host suitability of five tree species for the beetle. J. Forest Res..

[50] Maity S, Banerjee S, Biswas C, Guchhait R, Chatterjee A, Pramanick K (2021). Functional interplay between plastic polymers and microbes: a comprehensive review. Biodegradation.

[51] Shah AA, Kato S, Shintani N, Kamini NR, Nakajima-Kambe T (2014). Microbial degradation of aliphatic and aliphatic-aromatic *co*-polyesters. Appl. Microbiol. Biotechnol..

[52] Wei XF, Capezza AJ, Cui Y, Li L, Hakonen A, Liu B (2022). Millions of microplastics released from a biodegradable polymer during biodegradation/enzymatic hydrolysis. Water Res..

[53] Hoshino A, Isono Y (2002). Degradation of aliphatic polyester films by commercially available lipases with special reference to rapid and complete degradation of poly(L-lactide) film by lipase PL derived from *Alcaligenes* sp. Biodegradation.

[54] Nofal RM (2023). Biodegradable Textiles, Recycling, and Sustainability Achievement, Handbook of Biodegradable Materials.

[55] Kasirajan S, Ngouajio M (2012). Polyethylene and biodegradable mulches for agricultural applications: a review. Agron. Sustain. Dev..

[56] Todd EM, Zimmerman SC (2007). Supramolecular star polymers. Increased molecular weight with decreased polydispersity through self-assembly. J. Am. Chem. Soc..

[57] Sakai Y, Isokawa M, Masuda T, Yoshioka H, Hayatsu M, Hayano K (2002). Usefulness of soil *p*-nitrophenyl acetate esterase activity as a tool to monitor biodegradation of Polybutylene Succinate (PBS) in cultivated soil. Polym. J..

[58] Tsuboi S, Tanaka T, Yamamoto-Tamura K, Kitamoto H (2018). High-throughput method for the evaluation of esterase activity in soils. J. Microbiol. Methods.

[59] Tsuboi S, Yamamoto-Tamura K, Takada A, Yonemura S, Hoshino YT, Kitamoto H (2022). Selection of *p*-nitrophenyl fatty acid substrate suitable for detecting changes in soil esterase activity associated with degradation of biodegradable polyester mulch films: a field trial. Italian J. Agro..

[60] Yamamoto-Tamura K, Hiradate S, Watanabe T, Koitabashi M, Sameshima-Yamashita Y, Yarimizu T (2015). Contribution of soil esterase to biodegradation of aliphatic polyester agricultural mulch film in cultivated soils. AMB Express.

