# Supplementary material for: Dual Application of p-Nitrophenol Alkanoate-Based Assay for Soil Selection and Screening of Microbial Strains for Bioplastic Degradation
Source: J Microbiol Biotechnol. 2024 May 30;34(7):1530–43. doi: 10.4014/jmb.2403.03013 (PMC11294652; doi:10.4014/jmb.2403.03013)
Supplement: Supplementary file 1 [file jmb-34-7-1530-supple.pdf]

J. Microbiol. Biotechnol. 2024. 34(7): 1530–1543

https://doi.org/10.4014/jmb.2303.03013

Supplementary Figure

Dual Application of *p*-Nitrophenol Alkanoate-Based Assay for Soil Selection and Screening of Microbial Strains for Bioplastic Degradation

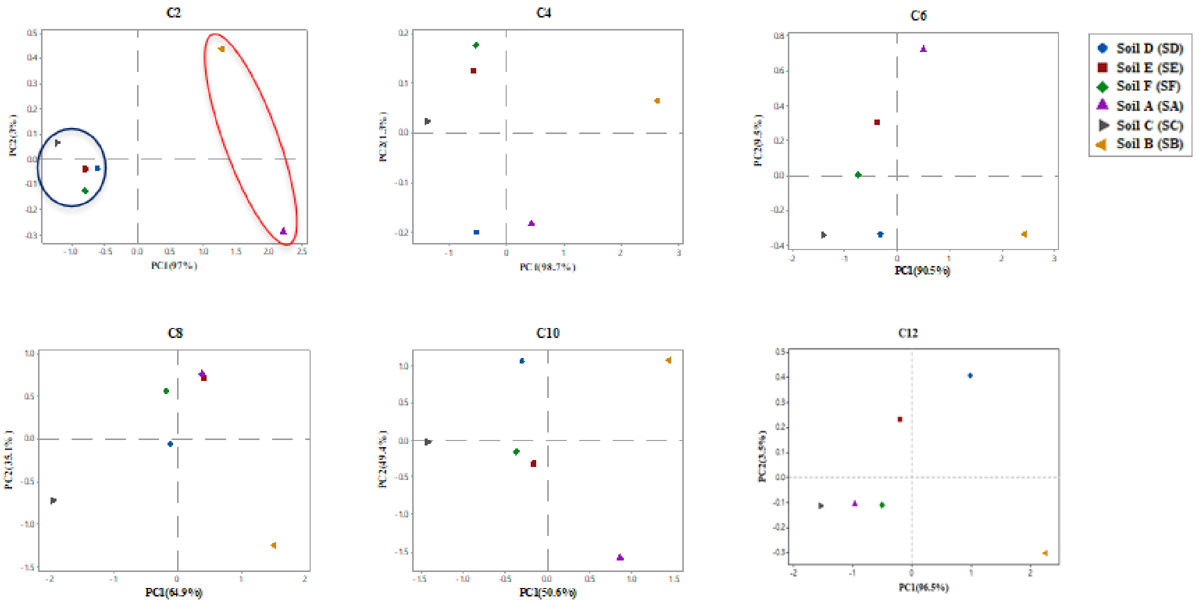

Fig. S1. PCA analysis for soil esterase activity test. PCA result for OD value after esterase activity test according to substrates (C2-C12).
